# Accuracy of an artificial intelligence as a medical device as part of a UK-based skin cancer teledermatology service

**DOI:** 10.3389/fmed.2024.1302363

**Published:** 2024-03-22

**Authors:** Helen Marsden, Polychronis Kemos, Marcello Venzi, Mariana Noy, Shameera Maheswaran, Nicholas Francis, Christopher Hyde, Daniel Mullarkey, Dilraj Kalsi, Lucy Thomas

**Affiliations:** ^1^Skin Analytics Ltd., London, United Kingdom; ^2^Blizard Institute, The Faculty of Medicine and Dentistry, Queen Mary University of London, London, United Kingdom; ^3^Chelsea and Westminster Hospital NHS Foundation Trust, London, United Kingdom; ^4^Imperial College Healthcare NHS Trust, St Mary's Hospital, London, United Kingdom; ^5^Exeter Test Group, Department of Health and Community Sciences, University of Exeter Medical School, Exeter, United Kingdom

**Keywords:** artificial intelligence, skin cancer, deep ensemble for the recognition of malignancy (DERM), teledermatology, AI as a medical device, skin analytics

## Abstract

**Introduction:**

An artificial intelligence as a medical device (AIaMD), built on convolutional neural networks, has demonstrated high sensitivity for melanoma. To be of clinical value, it needs to safely reduce referral rates. The primary objective of this study was to demonstrate that the AIaMD had a higher rate of correctly classifying lesions that did not need to be referred for biopsy or urgent face-to-face dermatologist review, compared to teledermatology standard of care (SoC), while achieving the same sensitivity to detect malignancy. Secondary endpoints included the sensitivity, specificity, positive and negative predictive values, and number needed to biopsy to identify one case of melanoma or squamous cell carcinoma (SCC) by both the AIaMD and SoC.

**Methods:**

This prospective, single-centre, single-arm, masked, non-inferiority, adaptive, group sequential design trial recruited patients referred to a teledermatology cancer pathway (clinicaltrials.gov NCT04123678). Additional dermoscopic images of each suspicious lesion were taken using a smartphone with a dermoscopic lens attachment. The images were assessed independently by a consultant dermatologist and the AIaMD. The outputs were compared with the final histological or clinical diagnosis.

**Results:**

A total of 700 patients with 867 lesions were recruited, of which 622 participants with 789 lesions were included in the per-protocol (PP) population. In total, 63.3% of PP participants were female; 89.0% identified as white, and the median age was 51 (range 18–95); and all Fitzpatrick skin types were represented including 25/622 (4.0%) type IV-VI skin. A total of 67 malignant lesions were identified, including 8 diagnosed as melanoma. The AIaMD sensitivity was set at 91 and 92.5%, to match the literature-defined clinician sensitivity (91.46%) as closely as possible. In both settings, the AIaMD identified had a significantly higher rate of identifying lesions that did not need a biopsy or urgent referral compared to SoC (*p*-value = 0.001) with comparable sensitivity for skin cancer.

**Discussion:**

The AIaMD identified significantly more lesions that did not need to be referred for biopsy or urgent face-to-face dermatologist review, compared to teledermatologists. This has the potential to reduce the burden of unnecessary referrals when used as part of a teledermatology service.

## Introduction

The global burden of skin cancer is growing, but healthcare systems lack the necessary capacity, especially in the aftermath of the COVID-19 pandemic. Skin cancers, primarily melanoma, squamous cell carcinoma (SCC), and basal cell carcinoma (BCC), are the most common cancers worldwide. In the United States, 9,500 people are diagnosed daily with annual treatment costs of $8.1bn (1). Skin cancer accounts for half of all cancers diagnosed in England and Wales and is increasing by 8% annually (2). However, of over 500,000 urgent referrals made to UK Secondary Care in 2019/20, only 6.5% resulted in a skin cancer diagnosis. Moreover, 25% of melanoma are found in non-urgent dermatology referrals (3) and diagnostic delays of 2 weeks or more can lead to a 20% decrease in 5-year survival rates (4). With approximately one in four UK Consultant Dermatologist posts unfilled (2), the situation is unsustainable.

A novel AI as a medical device (AIaMD), built on convolutional neural networks, has previously demonstrated high sensitivity for melanoma, similar to the level of skin cancer specialists (5). Trained using machine learning to recognise the most common malignant, premalignant, and benign skin lesions, the AIaMD analyses a dermoscopic image of a skin lesion and returns a suspected diagnosis of melanoma, SCC, BCC, Bowen’s disease/intraepidermal carcinoma (IEC), actinic keratosis (AK), atypical nevus (AN), or benign (labels of individual benign conditions are possible, but as the patient management is often the same, they are grouped into one output), along with a corresponding referral recommendation. The AIaMD applies a risk-based hierarchy so that the most serious potential diagnosis is returned. For example, if the AIaMD identifies a lesion as potentially either a BCC or melanoma, it will return a classification of melanoma.

The AIaMD is the key component of the Skin Analytics’ medical device deep ensemble for the recognition of malignancy (DERM), which is intended for use in the screening, triage, and assessment of skin lesions suspicious for skin cancer. DERM is deployed in the United Kingdom National Health Service (NHS) to support skin cancer diagnosis pathways that have assessed over 81,000 patients since 2020. After a period of use as a Class I device for clinical decision support, during which time this study was conducted, DERM received UKCA Class IIa approval in April 2022, allowing it to be used for autonomous decision-making, to further optimise the urgent referral pathways. To be of clinical value, the AIaMD needs to achieve a high specificity for premalignant and benign lesions as well as a high sensitivity for skin cancer. This study compared the rate and accuracy of the AIaMD and teledermatology in identifying premalignant and benign lesions that do not require biopsy or urgent referral while maintaining a high sensitivity for malignancy.

## Materials and methods

### Study design

This prospective, single-centre, single-arm, masked, non-inferiority design trial (the “Impact study”), with an adaptive group sequential design, was conducted at Chelsea and Westminster NHS Foundation Trust between February 2020 and August 2021. Chelsea and Westminster, which serves a population of 620,000 that has a demographic profile comparable with the London average (6), established an urgent skin cancer teledermatology service in 2017 (7) where patients with suspicious skin lesions can be referred from primary care.

The primary objective of this study was to demonstrate that the AIaMD had a higher rate of correctly classifying premalignant and benign lesions as not needing to be referred for biopsy or urgent face-to-face review compared to teledermatology standard of care (SoC) while achieving the same sensitivity to detect malignancy. Secondary endpoints included the sensitivity, specificity, positive and negative predictive values, number needed to biopsy for malignancy, and number needed to refer for premalignancy (IEC and AK) of the AIaMD and SoC. These performance data were used to conduct a simple cost impact assessment, based on the assumptions that teledermatology reviews cost £115.44 and require 10 min of specialist time per case on average; face-to-face assessments cost £163.41 and require 15 min per case; and biopsies cost £257.43 and require on average 32.5 min per lesion (based on a 50:50 split of excision biopsies which are booked for 45 min and incisional/punch biopsies which are booked for 20 min) (8, 9). This will be used to inform future health economic assessments. Surveys were conducted on patients’ perspectives on AIaMD use in their care and are reported in another publication (10). The study was registered on clinicaltrials.gov (NCT04123678) and was approved by the West Midlands-Edgbaston Research Ethics Committee and UK Health Research Authority on 23 December 2019.

### Participants

Patients aged 18 or over with at least one suspicious lesion being photographed as part of SoC were invited to consent to the study in a consecutive series. Patients who returned to the teledermatology service with the same or different lesions were able to re-consent to the study. To be eligible for inclusion in the study, lesions needed to be less than 15 mm in diameter (so as to fit within the dermatoscope lens); in an anatomical location suitable for photography (avoiding genital, hair-bearing, mucosal sites, and subungual sites), have no previous trauma including biopsy or excision; and have no visible scarring or tattooing.

### Procedures

In the teledermatology service, digital single-lens reflex (DSLR) and dermatoscope images of each suspicious lesion are taken by medical photographers. These images are reviewed remotely, alongside the primary care referral letter and patient-reported medical history, by consultant dermatologists who record a suspected diagnosis, and triage the lesion(s) for surgery, further assessment, or discharge.

Patients who consented to the study had an additional macroscopic and dermoscopic image of each lesion taken, using an iPhone XR smartphone and DermLite DL1 basic dermoscopic lens attachment, by a healthcare assistant (HCA). The suspected diagnosis and management decision recorded by the teledermatologist, and any subsequent patient review by skin cancer specialists, were collected, along with relevant medical history, patients’ levels of concern, healthcare resource utilisation data (number of appointments, time required to take images), and histopathology results where biopsies were undertaken. The iPhone XR dermoscopic images were used for AIaMD assessment, while the teledermatology review was conducted utilising DSLR images in accordance with the established SoC at Chelsea and Westminster. Patients completed all study-related activities in one visit, but the AIaMD image analysis occurred outside of the study, so clinicians were blinded to its output, and patient care was unaffected. Dermoscopic images were first quality-checked using an AI tool that assesses whether an image is dermoscopic, blurry, or dark, and rejected images were excluded from the AIaMD assessment.

### Statistics and analysis

Based on the reported prevalence of and dermatologist sensitivity for melanoma, SCC, and BCC (5, 11, 12), it was estimated that dermatologists would correctly identify 91.46% of skin cancers. The AIaMD settings were optimised to match this as closely as possible with the aim of achieving a difference of <0.2%. The closest AIaMD settings that could be achieved were 91% (AIaMD-A) and 92.5% (AIaMD-B). As both options were > 0.2% of the estimated dermatologist sensitivity, both settings were used for the primary endpoint. For the secondary endpoints, AIaMD-A was used as it was closer to the estimated clinical sensitivity.

The expected specificity of the AIaMD to identify malignancies was 54%, and the expected prevalence rates for MM, SCC, and BCC were 4.12, 5.16, and 21.39%, respectively. To demonstrate that the specificity of the AIaMD was not inferior to the specificity of SoC, using a 1% non-inferiority margin and with 99% power, a sample size of 634 lesions was needed. Assuming 1.2 lesions per patient and allowing for a 10% dropout rate, the sample size required was estimated to be 581 patients.

An interim analysis was conducted when the first third of data had been collected, to allow data-driven sample size reassessments. The primary endpoint was analysed using a one-sided, 2-proportion Z-test, with an overall alpha of 0.05. The final analysis was performed by combining the *p*-values from both phases of the study, using the procedure described by Lehmacher and Wassmer. The *p*-values of the test statistic from both phases of the study were therefore combined using specific predefined weights set as 0.577 and 0.82 for phases 1 and 2, respectively (13). The one-sided significance level was adjusted to 0.0246 for the final analysis based on the O’Brien-Fleming approach (14). Statistical estimates of accuracy are reported with 95% confidence intervals (CIs). Statistical analysis was conducted using R language version 4.1.3 (The R Project for Statistical Computing).

The suspected diagnosis and management outcome from the AIaMD and teledermatology were compared with a histologically confirmed diagnosis, where obtained, and failing that, consultant dermatologist diagnosis and management with a second opinion where available. Only histopathological diagnosis was accepted for melanoma, SCC, or BCC diagnosis, with a second review for melanoma. Final opinions on clinical diagnosis were provided by authors MN and LT, both consultant dermatologists, who also checked histopathology reports of all biopsied lesions and confirmed that no cases of rare skin cancer were identified. Patients and lesions that did not meet the inclusion criteria were excluded from the intention-to-treat population (ITT), as were those lesions without a final diagnosis available. Lesions with no AIaMD result available (missing dermoscopic images, and/or where these failed the image quality assessment) were excluded from the per-protocol (PP) population. The specificity of AIaMD was defined as the percentage of lesions diagnosed as IEC, AK, AN, or benign that were labelled as IEC, AK, AN, or benign by the AIaMD. The specificity of dermatologists was defined as the percentage of lesions diagnosed as IEC, AK, AN, or benign that the teledermatologist referred for a routine dermatologist appointment or discharged.

The COVID-19 pandemic began after the study had commenced recruitment and led to the reassessment, often downgrading, of patient management decisions. This was captured; however, the primary analysis is based on the original patient management decisions by the dermatologists. For the secondary analysis, diagnostic accuracy indices (sensitivity, specificity, predictive values) were calculated by evaluating the performance on lesions grouped as malignant vs. premalignant/benign. For instance, an SCC labelled as a melanoma would count as a true positive in the sensitivity calculation for both the AIaMD and clinical (SoC) diagnosis.

### Real-world settings

For comparative purposes, a *post-hoc* analysis was conducted using the same version of the AIaMD with threshold settings that were used in live deployments at the time of the study analysis. These targeted a higher sensitivity of >95% for melanoma and SCC and > 90% for BCC (AIaMD-RWS). Accuracy metrics including sensitivity and specificity were calculated using these settings, and the results are presented to provide more insight as to the impact of AIaMD if it had been a real-world deployment.

## Results

### Patient and lesion populations

**Figure 1:**
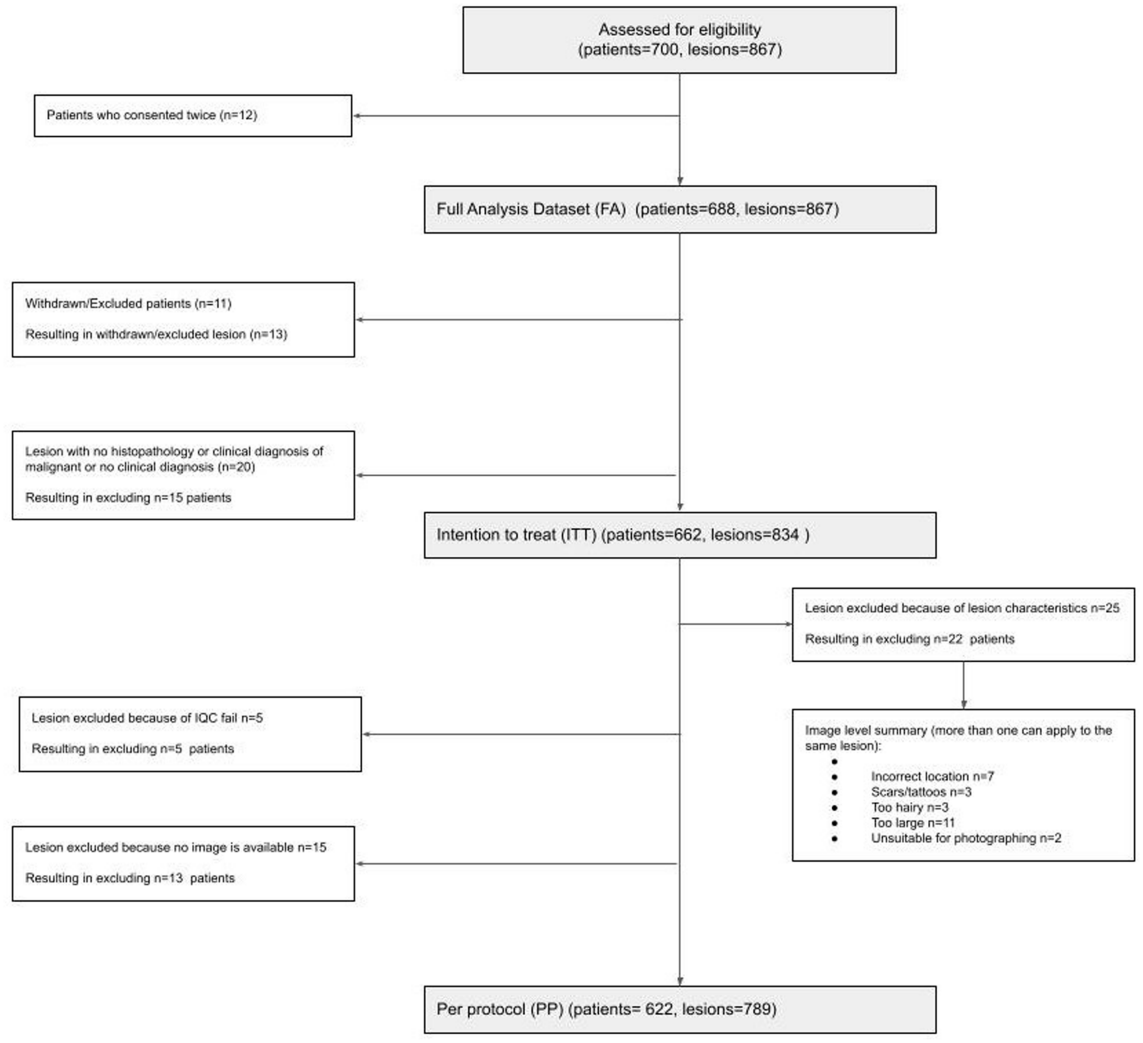
CONSORT flow diagram of study participants and lesions included in analyses.

A total of 688 participants (12 re-consented so 700 attendances) presenting with 867 lesions (average 1.3 lesions per patient) were recruited; 662 participants with 834 lesions were included in the intention-to-treat (ITT) population; and 622 participants with 789 lesions were included in the per-protocol (PP) population (Figure 1). In the PP population, 63.3% of participants were female; 89.0% identified as white, and the median age was 51 (range 18–95); and all Fitzpatrick skin types were represented including 25/622 (4.0%) type IV-VI skin (Table 1).

**Table 1:** Breakdown of the per-protocol patient population by age group, sex, ethnic group, Fitzpatrick skin type, and past personal history of skin cancer.

		*N*	%
Total number of patients		622	100
Age group	Mean	51.5	
	Standard deviation	19.6	
	Minimum	18	
	Maximum	95	
Sex	Female	394	63.3
	Male	228	36.7
Ethnic group	White	555	89.2
	Asian	14	2.3
	Black	8	1.3
	Other	10	1.6
	Mixed	22	3.5
	Unknown	13	2.1
Fitzpatrick skin type	I	203	32.6
	II	301	48.4
	III	93	15
	IV	12	1.9
	V-VI	13	2.1
Past personal history of skin cancer	None	490	78.8
	Melanoma	24	3.9
	SCC	10	1.6
	BCC	44	7.1
	Other	43	6.9
	Unknown	11	1.8

Most lesions were located on the face and scalp (25%), back (18.6%), arms (13.2%), and legs (19%) and had a history of change in the previous 3 months (86.3%). Lesions averaged 6.3 mm (range 0.5–15 mm) in diameter, and patients were most often (68.1%) a little concerned about their lesions (Table 2).

**Table 2:** Breakdown of per-protocol lesions by size in millimetres, body location, patient concern, and history of change.

		*N*	%
Lesion size (mm)	Mean	6.3	
	Standard deviation	3	
	Minimum	0.5	
	Maximum	15	
Lesion location	Face and scalp	197	25
	Neck	34	4.3
	Right arm	48	6.1
	Left arm	56	7.1
	Right palm	3	0.4
	Left palm	1	0.1
	Anterior chest	94	11.9
	Abdomen	54	6.8
	Posterior chest	3	0.4
	Back	147	18.6
	External genitals	1	0.1
	Right leg	72	9.1
	Right sole	0	0
	Left leg	78	9.9
	Left sole	1	0.1
Patient concern	Not concerned	94	11.9
	A little concerned	537	68.1
	Very concerned	144	18.3
	Unknown	14	1.8
Lesion change	None	108	13.7
	Changed colour	53	6.7
	More symptomatic	408	51.7
	New lesion	8	1
	Grown a bit	212	26.9
	Grown a lot	0	0

Sixty-seven malignant lesions were identified in the PP population: 8 melanoma, 13 SCCs, and 46 BCCs. Most melanomas were superficial spreading (*N* = 4) and < 1 mm thick (*N* = 7). Most SCCs were well or moderately differentiated (*N* = 9), while most BCCs were nodular (*N* = 22) (Table 3). Three additional lesions diagnosed as melanoma and four lesions diagnosed as SCCs had been included in the study but were ineligible because no images were available (1x melanoma, 1x SCC), the lesion was located on a scar (1x melanoma), or the lesions were larger than the dermoscopic lens (1x melanoma, 3x SCC).

**Table 3:** Breakdown of lesion diagnosis from histology or clinical diagnosis in the per-protocol population, including subtypes of malignant lesions, Breslow thickness of melanoma, and staging of squamous cell carcinoma and basal cell carcinoma.

			Clinical diagnosis	Histopathology
Total number of lesions			789	240
Malignant	(Suspect) melanoma		26	8
	Subtype	*In situ*		2
		Lentigo maligna		2
		Superficial spreading		4
		Nodular		0
	Breslow thickness	*In situ*		4
		<1.0 mm		3
		1.01–2.0 mm		1
		>2.0 mm		0
	(Suspect) squamous cell carcinoma		41	13
	Subtype	Well differentiated		4
		Moderately differentiated		5
		Poorly differentiated		0
		Unknown		4
	Stage	T1		6
		T2		1
		T3		0
		T4		1
		Unknown		5
	(Suspect) basal cell carcinoma		51	46
	Stage	Tis		1
		T1		20
		Unknown		25
	Subtype	Superficial		6
		Nodular		21
		Infiltrative		6
		Micronodular		1
		Unknown		12
Premalignant or benign	IEC/SCC *in situ*		21	7
	Actinic keratosis		35	25
	Atypical/dysplastic nevus		29	10
	Seborrheic keratosis		168	26
	Dermatofibroma		16	3
	Vascular lesion		13	4
	Lentigo		14	3
	Benign melanocytic Nevus		259	39
	Other (Benign)/unknown		116	56

### Primary outcome

The interim analysis of phase 1 included 199 lesions (21 malignant and 178 premalignant or benign). AIaMD-A correctly identified 77.5% of the premalignant and benign lesions (138/178, 95% CI 70.6–83.3%) as lesion types that did not need a biopsy or urgent face-to-face assessment and AIaMD-B identified 74.7% (133/178, 95% CI 67.6–80.8%) compared to 73.6% (131/178, 95% CI 66.4–79.8%) by SoC. The interim analysis of the primary endpoint confirmed the non-futility of the study; however, the required sample size increased to 700 patients, to achieve a statistical power of 95%. In phase 2, there were 590 lesions (46 malignant and 544 premalignant or benign). AIaMD-A correctly identified 85.1% of the premalignant and benign lesions (463 out of 544, 95% CI 81.8–87.9%) as lesions types that did not need a biopsy or urgent face-to-face assessment, and AIaMD-B identified 81.6% (444 out of 544, 95% CI 78.8–84.7%), compared to 71.3% by SoC (388 out of 544, 95% CI 67.3–75.1%). After weighing the two phases across the whole study as described in the Statistics and Analysis methods, AIaMD-A and AIaMD-B had a significantly higher rate of correctly identifying premalignant and benign lesions as lesions that did not need a biopsy or urgent face-to-face assessment compared to SoC (*p*-value<0.0246).

### Secondary outcomes

The sensitivity, positive and negative predictive values, and false negative and positive rates, of the teledermatologists and AIaMD to identify malignant lesions, were calculated (Table 4).

**Table 4:** Comparison of the accuracy of the standard of care (SoC) and artificial intelligence (AIaMD) for skin cancer detection in the per-protocol population.

	Sensitivity (%, 95 CI)	Specificity (%, 95 CI)	PPV (%, 95 CI)	NPV (%, 95 CI)	FNR (%, 95 CI)	FPR (%, 95 CI)	NNB (N, 95% CI)	NNR (N, 95% CI)
SoC	97.0, 88.7–99.5	71.9, 68.4–75.1	24.2, 19.3–29.9	99.6, 98.5–99.9	3.0, 0.5–11.3	28.1, 24.9–31.6	4.2 (3.3–5.5)	8.6 (6.2–12.3)
AIaMD	91.0, 80.9–96.3	83.2, 80.3–85.9	33.5, 26.8–40.9	99.0, 97.7–99.6	9.0, 3.7–19.1	16.8, 14.1–19.7	3 (2.4–3.7)	4.5 (3.6–5.8)

CI, confidence interval; PPV, positive predictive value; NPV, negative predictive value; FNR, false negative rate; FPR, false positive rate; NNB, number needed to biopsy to confirm a diagnosis of skin cancer; NNR, number needed to refer to confirm a diagnosis of IEC or AK; IEC, intraepidermal carcinoma (Bowen’s disease); AK, actinic keratosis.

**Figure 2:**
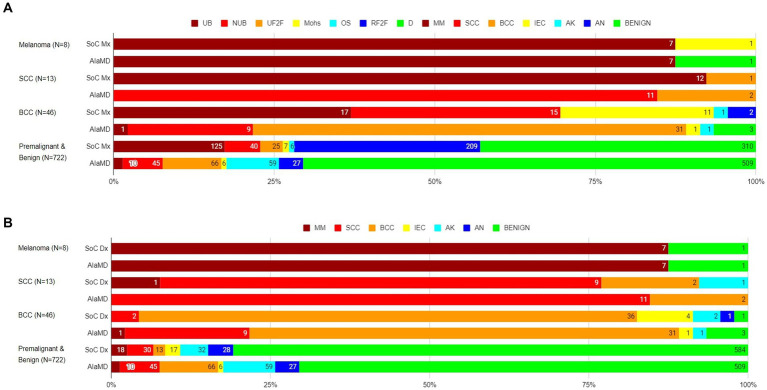
Standard of care management **(A)** and diagnosis **(B)** and artificial intelligence (AIaMD) classification of lesions compared to final diagnosis by histopathology or clinical diagnosis, in the per-protocol population. Standard of care management (SoC Mx): UB, urgent biopsy; NUB, non-urgent biopsy; UF2F, urgent face-to-face appointment; Mohs, BCC/Mohs clinic; OS, other specialty; RF2F, routine face-to-face appointment; D, discharge. Standard of care diagnosis (SoC Dx)/AIaMD label: MM, melanoma; SCC, squamous cell carcinoma; BCC, basal cell carcinoma; IEC, intraepidermal carcinoma; AK, actinic keratosis; AN, atypical nevus.

Of the 8 histology-diagnosed melanomas, seven were sent for urgent biopsy and one was referred to BCC/Mohs clinic by the teledermatologist. Seven were labelled as melanoma by both SoC and AIaMD, while the other melanoma was thought to be a traumatised angioma by SoC and was classified as benign by AIaMD. Of the 13 histology-confirmed SCCs, all 13 were sent to urgent biopsy or urgent face-to-face dermatologist appointment by SoC, 9 with a suspected diagnosis of SCC; and 12 were labelled SCC and 1 was labelled BCC by AIaMD. Of the 46 histology-confirmed BCCs, 43 were sent for biopsy or were referred to BCC/Mohs clinic by the teledermatologist, while 2 lesions were referred to routine face-to-face dermatology; 38 had suspected diagnoses from teledermatology of melanoma or BCC, while the remaining 8 lesions were referred with a suspected premalignant or benign diagnosis; and 31 were labelled as BCC, 11 as melanoma or SCC, and 4 as premalignant or benign by the AIaMD (Figure 2).

In total, 216 lesions were referred directly from teledermatology SoC to urgent or non-urgent biopsy. The number needed to biopsy (NNB) for SoC to diagnose one malignancy was 4.2 (216/51, 95% CI 3.3–5.5). If all lesions classified as malignant by the AIaMD were biopsied, the NNB was 3 (182/61, 95% CI 2.4–3.7) (Table 4).

A total of 268 lesions were referred from teledermatology SoC to biopsy or urgent face-to-face assessment. The number needed to refer (NNR) for SoC to diagnose one case of IEC or AK was 8.6 (268/31, 95% CI 6.2–12.3). If all lesions classified as malignant or premalignant by the AIaMD were referred, the NNR for IEC and AK was 4.5 (249/55, 95% CI 3.6–5.8) (Table 4).

SoC required 688 teledermatology patient reviews, 221 face-to-face assessments, and up to 299 lesion biopsies (240 biopsies were conducted with the missing biopsies mainly due to delays from ongoing pressures following the COVID-19 pandemic meaning histopathology reports were not available within the study data collection window). If lesions had been triaged in accordance with the AIaMD output, 454 patient reviews would not have been required on the skin cancer pathway, 141 face-to-face assessments would have been avoided, and 124 fewer lesions would have been biopsied. This equates to cost savings of £52,409.76 in teledermatology reviews, £23,040.81 in face-to-face assessments, and £31,921.32 in biopsies. In terms of specialist time, this would save 76 h in teledermatology reviews, 35 h of face-to-face appointments, and 67 h of biopsies. In total, this amounts to a cost impact of £107,371.89 and 178 specialist h saved. Extrapolated to per 1,000 patients entering the pathway, this would scale to £156,063.79 and 259 specialist hours saved.

Out of 867 lesions included in the study, 843 (97.2%) had dermoscopic images successfully captured, and 24 lesions could not be imaged dermoscopically using the iPhone X. In total, 837 dermoscopic images (99.3% of those captured) passed the image quality check, and it took the HCA an average of 1 min to capture the study images. No adverse events were reported in the study.

### *Post-hoc* analysis of real-world settings

The sensitivity, positive and negative predictive values, and false negative and positive rates, of the AIaMD-RWS, were also calculated (Table 5).

**Table 5:** Comparison of the accuracy of the standard of care (SoC) and artificial intelligence with real-world setting (AIaMD-RWS) for skin cancer detection in the per-protocol population.

	Sensitivity (%, 95 CI)	Specificity (%, 95 CI)	PPV (%, 95 CI)	NPV (%, 95 CI)	FNR (%, 95 CI)	FPR (%, 95 CI)	NNB (N, 95% CI)	NNR (N, 95% CI)
SoC	97.0, 88.7–99.5	71.9, 68.4–75.1	24.2, 19.3–29.9	99.6, 98.5–99.9	3.0, 0.5–11.3	28.1, 24.9–31.6	4.2 (3.3–5.5)	8.6 (6.2–12.3)
AIaMD-RWS	94, 84.7–98.1	73.3, 69.9–76.4	24.6, 19.6–30.4	99.2, 98–99.8	6, 1.9–15.3	26.7, 23.6–30.1	4.1 (3.3–5.1)	5.2 (4.1–6.6)

CI, confidence interval; PPV, positive predictive value; NPV, negative predictive value; FNR, false negative rate; FPR, false positive rate; NNB, number needed to biopsy to confirm a diagnosis of skin cancer; NNR, number needed to refer to confirm a diagnosis of IEC or AK; IEC, intraepidermal carcinoma (Bowen’s disease); AK, actinic keratosis.

**Figure 3:**
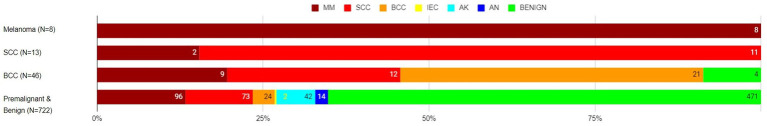
Artificial intelligence with real-world setting (AIaMD-RWS) classification of lesions compared to final diagnosis by histopathology or clinical diagnosis, in the per-protocol population. AIaMD-RWS: MM, melanoma; SCC, squamous cell carcinoma; BCC, basal cell carcinoma; IEC, intraepidermal carcinoma; AK, actinic keratosis; AN, atypical nevus.

Of the eight histology-diagnosed melanoma, the RWS-AIaMD correctly identified all eight as melanoma. Of the 13 histology-confirmed SCCs, 11 were correctly labelled as SCC by the RWS-AIaMD, with the remaining 2 classified as melanoma. Of the 46 histology-confirmed BCCs, 21 were labelled as BCC by the RWS-AIaMD, 21 as melanoma or SCC, and 4 as benign (Figure 3).

If all lesions classified as malignant by the AIaMD-RWS were biopsied, the NNB was 4.1 (256/63, 95% CI 3.3–5.1). If all lesions classified as malignant or premalignant by the AIaMD were referred, the NNR for IEC and AK was 5.2 (300/58, 95% CI 4.1–6.6).

## Discussion

This study demonstrates a high specificity for skin cancer of the AIaMD with a significantly lower rate of premalignant and benign lesion referral for biopsy or urgent face-to-face dermatologist review compared to SoC. AIaMD, therefore, shows potential to improve healthcare resource utilisation (HRU), which will be the subject of further health economic analyses utilising the data from this study. Assuming premalignant or benign AIaMD outputs meant that no further patient management on the urgent suspected cancer pathway was required, there could have been savings of >£100,000 and > 150 h of specialist time. There are, however, many other costs and benefits, as well as the potential need to expedite treatment for non-cancerous dermatological conditions, that should be considered when conducting health economic modelling.

While the high specificity of the AIaMD has the potential to improve HRU, this does raise the key question of the possible risk of a trade-off in sensitivity. The study-specific settings used by the AIaMD were determined to match an expected sensitivity by clinicians of 91.46%, which had been determined by a review of the literature on clinician sensitivity for skin cancer detection (5, 11, 12). The sensitivity achieved by teledermatologists in this study was higher than expected and higher than the sensitivity of the AIaMD to identify malignancies, either when used with the study-specific settings or the settings optimised for live deployment. This may be because the study was carried out at a centre with a well-established teledermatology service and experienced teledermoscopy clinicians, which is unlikely to be representative of UK dermatology more widely, as many centres have yet to implement urgent cancer teledermatology pathways. It is also important to note that the malignant lesions that the AIaMD missed were mostly BCC lesions. One lesion diagnosed as melanoma was classified as benign by the AIaMD with the study-specific settings but correctly identified when the live-deployment settings (AIaMD-RWS) were used, which also had a benign suspected clinical diagnosis by teledermatology, indicating the lesion was difficult to diagnose without a biopsy and that the AIaMD-RWS would have expedited treatment for the melanoma over and above SoC.

Furthermore, patient management being determined by teledermatology means that there was a risk of validation bias towards the outcome that validates the teledermatologist management plan (15). There were 25 lesions discharged by teledermatology but classified as malignant by the AIaMD (35 with RWS-AIaMD) that were not followed up, due to the length of time between patient recruitment into the study and image analysis by the AIaMD. This means there may have been malignant lesions that the AIaMD identified but the teledermatologist discharged. Of the patients who presented at the teledermatology service, and consented to the study, twice, three had a different clinical diagnosis at the second assessment, and two patients (four lesions) were subsequently biopsied. In all but one of the lesions reviewed twice, the AIaMD output was benign for the first assessment and only changed for the four lesions subsequently biopsied, indicating that the AIaMD was picking up similar features in the second assessment that prompted the clinicians to refer for a biopsy. Though beyond the scope of this study, the potential of malignant lesions missed by SoC, but identified by the AIaMD, should be considered for future research, as should the impact of changes in a lesion on the AIaMD classification.

This study builds on previous studies, which found that the AIaMD component of DERM can detect melanoma and non-melanoma skin cancer with accuracy comparable to specialists (5, 16–18), by looking at its accuracy to detect premalignant or benign lesions. The Melanoma Image Analysis Algorithm (MIAA) study evaluated the AIaMD on lesions that dermatologists referred for biopsy or were obviously benign (5), missing out those that GPs were concerned about but a dermatologist could diagnose and manage without a biopsy. While the non-melanoma skin cancer (NMSC) study included suspicious skin lesions that were not referred for a biopsy, the lesions were all assessed by a dermatologist prior to inclusion in the study, again missing lesions that look suspicious to a non-skin cancer specialist, but which a dermatologist is less concerned about (17, 18). The lesion population in this study was primarily based on lesions that had not first been evaluated by a dermatologist and is therefore more representative of the population that the AIaMD may be used on in primary care.

This study was conducted in a single site in North West London with a younger and more ethnically diverse population than the UK overall (6). The incidence of melanoma in the region is half of the national rate of (14 vs. 28 per 100,000) (19). There is a growing body of evidence that shows a drop in AI performance between research and real-world environments (20, 21). This means caution is needed in extrapolating these results, particularly the NPV, PPV, and NNB, into other settings in which the patient population, incidence and risk of skin cancer, and physician experience are different. The AIaMD has, however, been safely deployed in real-world pathways by incorporating clinical reviews of its outputs. Indeed, real-world evidence of AIaMD performance continues to be collated showing strong performance and impact (22–26). There is an ongoing study to optimise how it is integrated into clinical pathways and workflows, as well as to evaluate the real-world impact with respect to health economics (27, 28).

The Get-It-Right-First-Time (GIRFT) dermatology workforce recommendations include the uptake of digital technologies to achieve more efficient NHS HRU (29). Implementing AIaMD services could allow trusts and dermatologists to dedicate more time to meeting skin cancer targets; individual cancer patients; addressing the post-pandemic backlog; patients with other severe skin diseases requiring systemic/biologic medication; and teaching/research. Moreover, this could reduce clinician burnout and increase the recruitment/retention of dermatologists with a greater capacity for trainees to see cases in addition to skin cancer referrals. Most importantly, this offers the opportunity to reduce reliance on insourcing and waitlist initiatives, which are short-term solutions to deep-seated and long-term dermatology capacity issues.

The UK Faster Diagnosis Standard (FDS) is currently being implemented, with a target of communicating to patients referred on cancer pathways their diagnosis within 28 days (30). As of June 2023, approximately one in every five NHS trusts was not able to meet this target for skin cancer referrals (31). The immediacy of AIaMD outputs allows for quicker communication of premalignant and benign lesion classifications as well as the potential for greater surgical capacity to ensure more timely biopsies.

In the US, the American Academy of Dermatology supports skin cancer screenings at community events (32). An action in the FDS is to ‘consider linking the development of Community Locality Image Centres to Community Diagnostic Centres, to provide high-quality images for teledermatology and teledermoscopy activity’ (30). Given almost all lesions in this study were photographed by an HCA within 1 min, both settings could use the AIaMD to provide faster access to care in more remote locations, furthering the potential HRU benefits from fewer patients being unnecessarily referred for specialist assessment.

A recent UK government report highlighting several projects evaluating AI within healthcare stated that there are currently no standardised methods for the real-world evaluation of AI. Independent evaluations of the DERM service are ongoing, but a description of a real-world deployment of DERM at University Hospitals Birmingham is noted to have ‘helped 40% of patients avoid the need for a hospital appointment’ (33). There are also examples in other therapy areas where AI might increase the speed of diagnosis [e.g., lung cancer (34) and heart failure (35)], but data in this regard are limited and an assessment of their impact on HRU is not yet available.

The COVID-19 pandemic impacted the study: Recruitment was suspended during national lockdowns; 48 patients had their management changed, usually downgraded; and follow-up appointments and non-urgent biopsies were delayed, including some biopsies that occurred more than 3 months after AIaMD assessment (no malignancies were identified in these). Indeed, the final diagnoses of 40 lesions could not be confirmed by biopsy or face-to-face assessment within the timeframe of the study. For these, LT conducted a second teledermatology assessment to provide a final diagnosis. Furthermore, elderly and immunosuppressed patients, who are at high risk of both COVID-19 and skin cancer, were encouraged to isolate during the pandemic and may have delayed seeking medical care during this time, which might account for the lower-than-expected incidence of malignancy in the study.

Connectivity issues led to some initial image-capture difficulties, but very few lesions had no images captured or failed image quality assessment, indicating an improvement in the image-capture process used in the MIAA study (5). This is likely to be due to the accessibility of capturing images using smartphones rather than a DSLR camera, emphasised by the images being captured in a minute on average. Technological deployment issues do remain a challenge that must be addressed for successful real-world deployment of the AIaMD.

There were no rare skin cancers identified in this study. This is not unexpected given the low incidence of rare skin cancers (3.1 per 100,000, 95% CI 3.0–3.2, in the UK in 2018–2020) and even lower incidence of specific rare skin cancers (e.g., 0.62, 95% CI 0.58–0.66 for Merkel cell carcinoma in the UK in 2018–2020) as opposed to skin cancer as a whole (387 per 100,000, 95% CI 386–388) (36). A few cases of rare skin cancers have been included in other studies of AIaMD (18, 26); however, additional data are needed to demonstrate the performance of the AIaMD.

The study was also reflective of the low incidence of skin cancers in higher Fitzpatrick skin types across a large population; however, it was not large enough to identify any malignant lesions in patients with darker skin, nor, therefore, to demonstrate the performance of the AIaMD in these patients. This is again to be expected given that less than 0.5% of skin cancers diagnosed in the UK are in Black and Asian patients (37). These cases often present late or are missed in the conventional care setting, making it difficult to demonstrate the performance of a novel product in patient groups with a low incidence of skin cancer through classical clinical studies. Efforts are ongoing to improve datasets in these under-represented patient groups, including surveillance of deployments and international collaborations.

AI systems can suffer from overfitting, hindering generalisability (38). The AIaMD algorithm has been trained on dermoscopic images of skin lesions from multiple sources. Biases may exist in these datasets, reducing AIaMD performance in different populations; however, the accuracy of the AIaMD observed is similar to previous reports (5, 16–18), demonstrating limited overfitting and good generalisability across novel datasets. Only one smartphone and dermatoscope combination was used, which is different from previous studies (5, 16–18), so no direct comparison of AIaMD performance on images captured by different devices can be made. This is controlled in real-world deployments of AIaMD too, however, whereby specific combinations of smartphones and dermatoscopes are qualified for usage with AIaMD, which is a mechanism of standardising the input to support consistent performance.

Finally, while the MIAA, NMSC, and impact studies show the performance of this particular AIaMD, these results cannot be generalised to the potential impact of other AI-based skin cancer detection tools. Indeed, a study of 25 freely downloadable AI apps found an average sensitivity of <30% for melanoma (39); a multicentre trial across Australia and Austria of a mobile phone-based AI found its performance was significantly inferior to specialists in a real-world scenario (40); and an AI system studied in Canada identified 6 out of 10 melanoma included in the study (41). Importantly, the AIaMD evaluated here is a component of the first and, at the time of writing, only AI-based skin cancer detection product that is a Class IIa UKCA Medical Device. This is crucial not only as a verification of safety from the Medicines and Healthcare products Regulatory Agency (MHRA) but also for all of the systems in place to monitor and improve the technology. This certification opens up further opportunities for AIaMD to triage patients with skin lesions to the most appropriate next step with the aim of improved access and early diagnosis for all patients with suspected skin cancer.

## Data availability statement

The raw data supporting the conclusions of this article will be made available by the authors, without undue reservation.

## Ethics statement

The studies involving humans were approved by West Midlands – Edgbaston Research Ethics Committee and UK Health Research Authority. The studies were conducted in accordance with the local legislation and institutional requirements. The participants provided their written informed consent to participate in this study.

## Author contributions

HM: Conceptualization, Data curation, Formal analysis, Funding acquisition, Investigation, Methodology, Project administration, Resources, Software, Supervision, Validation, Visualization, Writing – original draft, Writing – review & editing. PK: Data curation, Formal analysis, Investigation, Methodology, Software, Validation, Writing – review & editing. MV: Data curation, Formal analysis, Investigation, Methodology, Software, Validation, Writing – review & editing. MN: Data curation, Investigation, Validation, Writing – review & editing. SM: Data curation, Investigation, Project administration, Validation, Writing – review & editing. NF: Data curation, Validation, Writing – review & editing. CH: Data curation, Methodology, Visualization, Writing – review & editing. DM: Data curation, Investigation, Project administration, Resources, Supervision, Writing – review & editing. DK: Data curation, Formal analysis, Investigation, Project administration, Validation, Visualization, Writing – original draft, Writing – review & editing. LT: Conceptualization, Data curation, Formal analysis, Funding acquisition, Investigation, Methodology, Project administration, Resources, Supervision, Validation, Visualization, Writing – original draft, Writing – review & editing.
