# Supplementary material for: Accuracy of an artificial intelligence as a medical device as part of a UK-based skin cancer teledermatology service
Source: Front Med (Lausanne). 2024 Mar 22;11:1302363. doi: 10.3389/fmed.2024.1302363 (PMC10996444; doi:10.3389/fmed.2024.1302363)
Supplement: Supplementary file 1 [file Table_1.DOCX]

Supplementary Table 1: Breakdown of “Other” diagnoses, as determined by histopathology or clinical diagnosis.

| Diagnosis source | Diagnosis | Count (N) |
| --- | --- | --- |
| Histopathology | Apocrine Cystadenoma | 1 |
|  | Benign Keratosis | 2 |
|  | Benign Squamoproliferative | 1 |
|  | Cartilage | 1 |
|  | Chalazion | 1 |
|  | Chondrodermatitis Nodularis Helicis | 1 |
|  | Cyst | 2 |
|  | Granuloma Annulare | 1 |
|  | Granulomatous Inflammation | 1 |
|  | Hypertrophic Lichen Planus | 1 |
|  | Inflammation | 1 |
|  | Juvenile Xanthogranuloma | 1 |
|  | Keratoacanthoma | 7 |
|  | Keratosis | 1 |
|  | Large Cell Acathoma | 1 |
|  | Lichenoid Inflammation | 3 |
|  | Psoriasiform Dermatitis | 1 |
|  | Pyogenic Granuloma | 2 |
|  | Ruptured Follicle | 1 |
|  | Scar | 2 |
|  | Sebaceous Hyperplasia | 1 |
|  | Skin Tag | 1 |
|  | Stasis Dermatitis | 1 |
|  | Subacute Spongiotic Dermatitis | 1 |
|  | Sun Damage | 1 |
|  | Syringoma | 1 |
|  | Tricholemmoma | 1 |
|  | Ulceration | 1 |
|  | Unknown | 4 |
|  | Venous Stasis | 1 |
|  | Venous Stasis Dermatitis | 1 |
|  | Viral Wart | 10 |
| Clinical Diagnosis | Ak | 6 |
|  | Benign Melanocytic Lesion | 3 |
|  | Blue Naevus | 1 |
|  | Comedone | 3 |
|  | Cystic / Ingrowing Hair | 1 |
|  | Cyst | 5 |
|  | Dermatosis Papulosa Nigra | 1 |
|  | Fibrous Papule | 1 |
|  | Inflammatory | 3 |
|  | Lichenoid Keratosis | 10 |
|  | Lipoma Or Cyst | 2 |
|  | Meyersson Naevus | 1 |
|  | Normal Skin | 1 |
|  | Onychomycosis | 1 |
|  | Probably Sebaceous Hyperplasia | 1 |
|  | Scar | 2 |
|  | Sebaceomas | 1 |
|  | Solar Lentigo | 2 |
|  | Spider Naevus | 1 |
|  | Telagiectasia | 1 |
|  | Unknown | 11 |
|  | Venous Stasis | 2 |
|  | Viral Wart | 6 |
